# Supplementary material for: The Ethylene Response Factor ERF5 Regulates Anthocyanin Biosynthesis in ‘Zijin’ Mulberry Fruits by Interacting with MYBA and F3H Genes
Source: Int J Mol Sci. 2022 Jul 9;23(14):7615. doi: 10.3390/ijms23147615 (PMC9318412; doi:10.3390/ijms23147615)
Supplement: Supplementary file 1 [file ijms-23-07615-s001.zip › Supp Figures.pdf]

Supplementary Figures

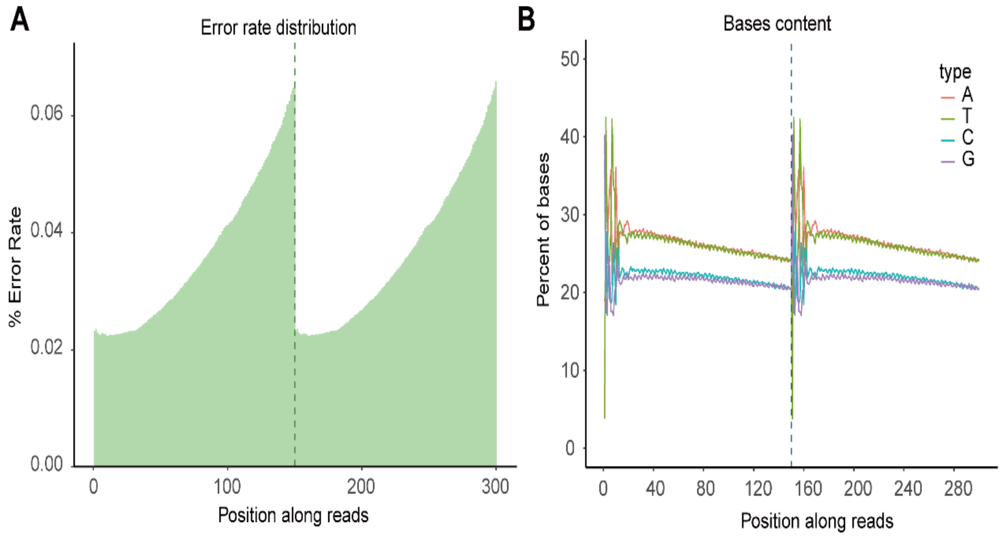

Figure S1. Quality control (QC) of RNA sequencing data across the two mulberry genotypes, Zijing (ZJ) and Dashi (DS). **A**, Error rate distribution against the reads position; **B**, distribution of nucleotide bases (A, T, C and G) in two mulberry genotypes along the reads positions.

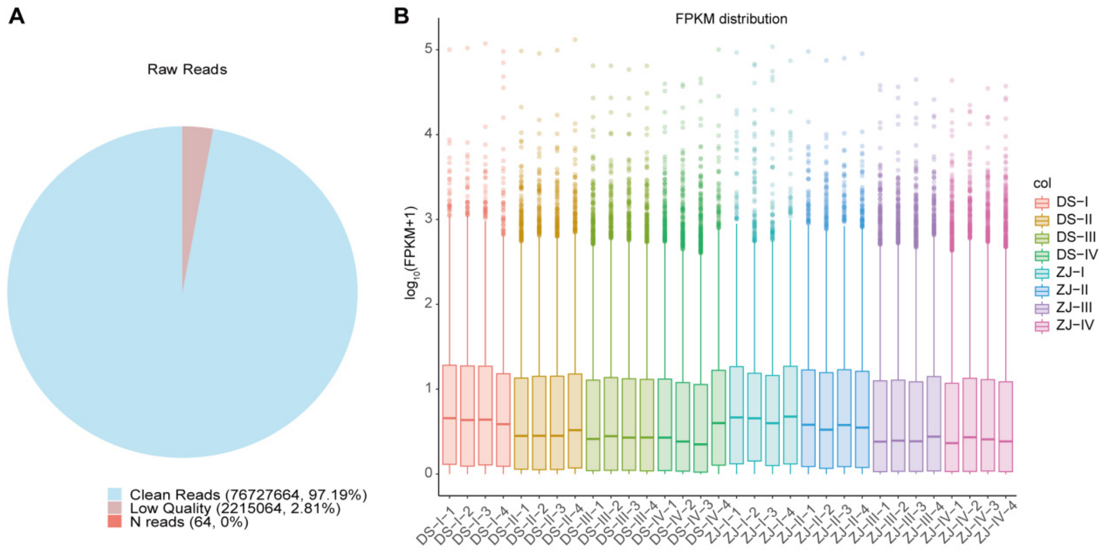

Figure S2. Distribution of raw and clean reads of the transcriptomic dataset in DS and ZJ mulberry genotypes at four different fruit developmental stages. **A**, raw reads distribution; **B**, Fragments Per Kilobase of transcript per Million mapped reads (FPKM) values for each sample. Four biological replicates were performed for each mulberry genotype and four technical replicates for each biological replicate.

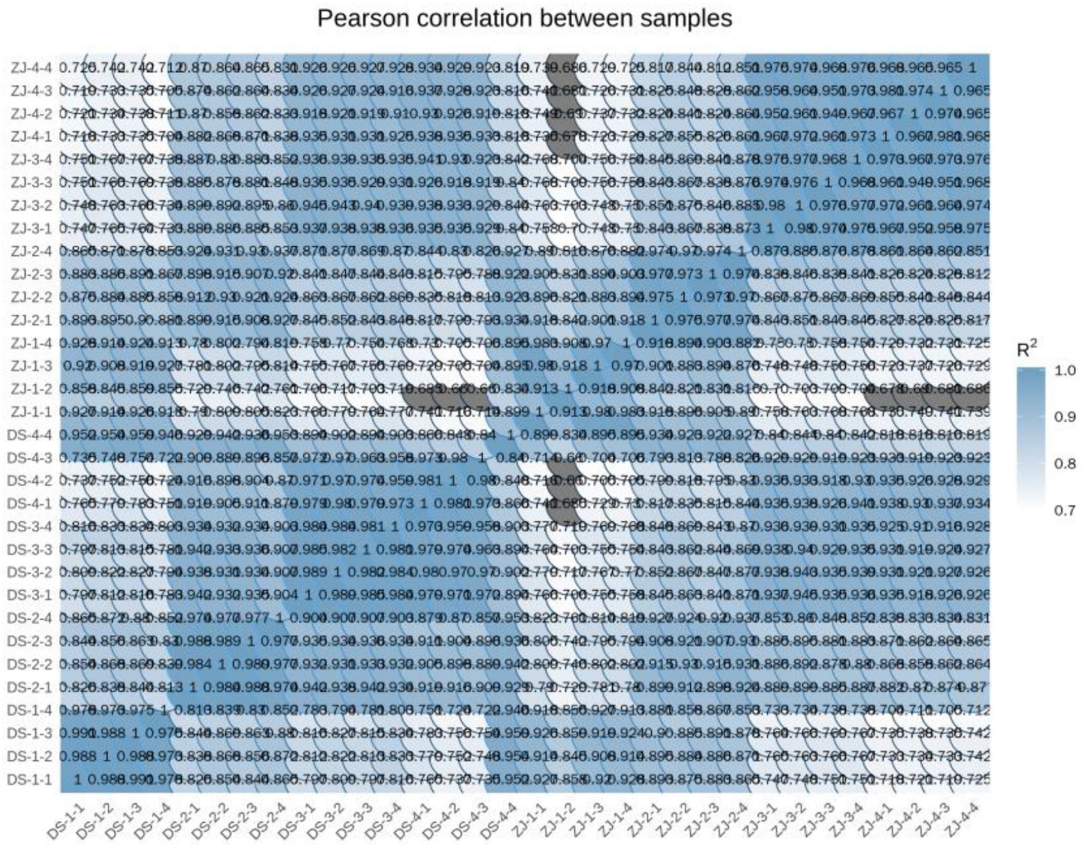

Figure S3. Evaluation of relatedness degree between the different samples for two mulberry genotypes (ZJ and DS) based on transcriptome dataset. The different colors in the cells represent the increasing Pearson correlation coefficient (PCC) from light (R^2=0.7) to dark blue (R^2=1). The number following each mulberry genotype indicates different stages accordingly.

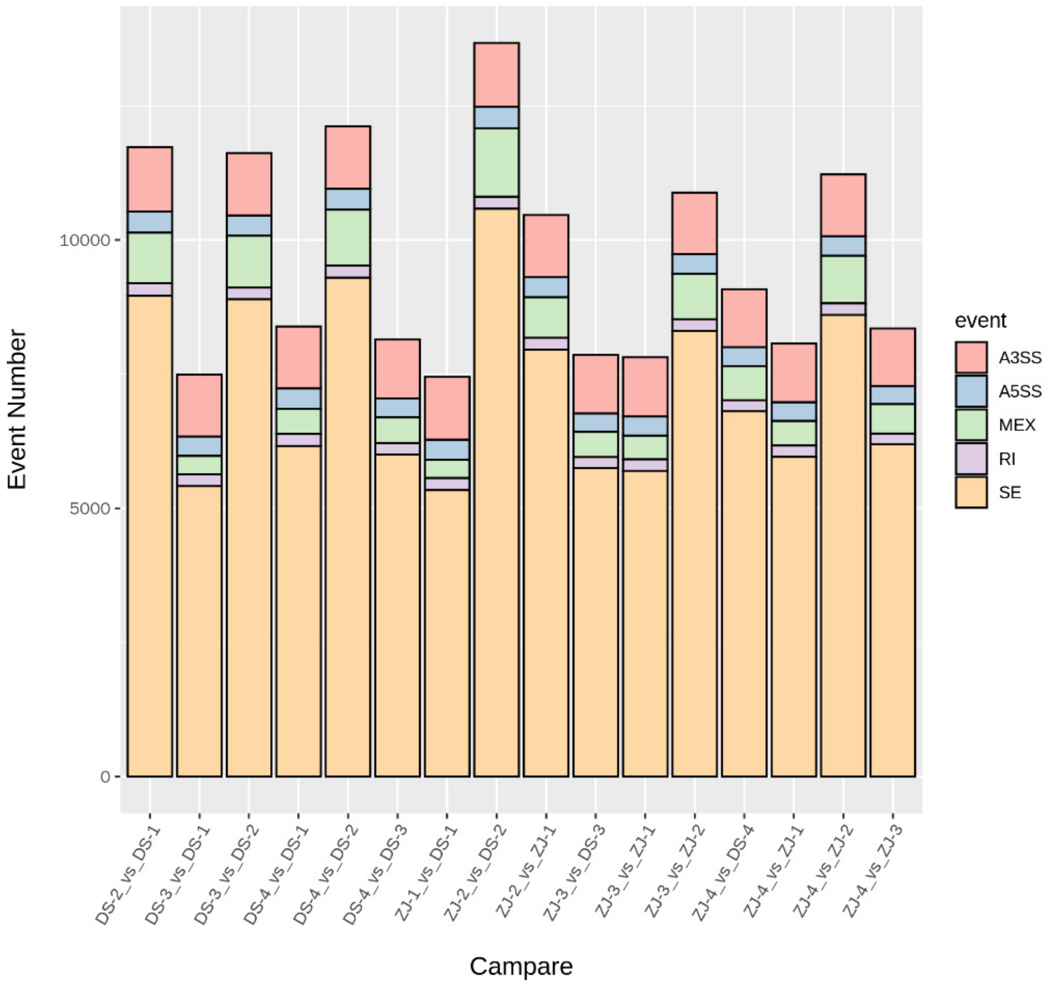

Figure S4. Differentiation analysis on the events of alternative splicing (AS) for the two mulberry genotypes ZJ and DS. Abbreviations: SE: Skipped exon; MXE: Mutually exclusive exon; A5SS: Alternative 5' splice site; A3SS: Alternative 3' splice site; RI: Retained intron. The number following each mulberry genotype indicates different stages accordingly.

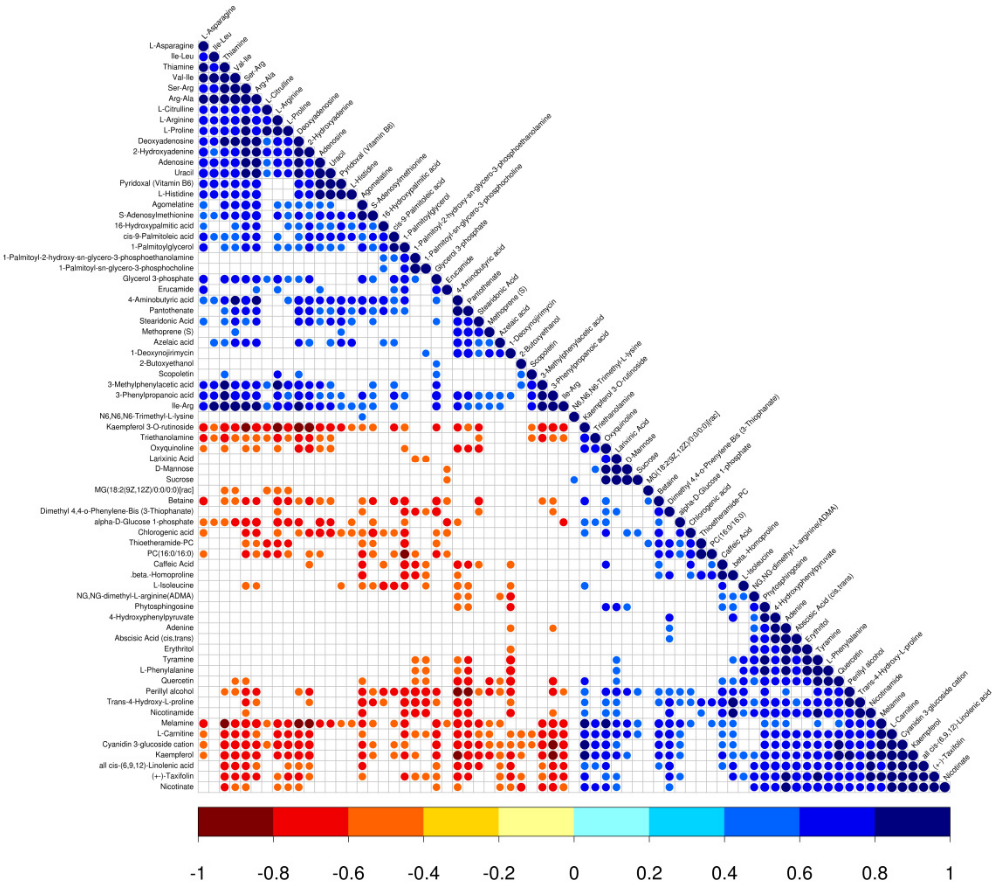

Figure S5. Correlation of non-targeted metabolites across the two mulberry genotypes ZJ and DS. The Pearson correlation coefficient (PCC) was indicated in different colors in the cells, which varies between -1 to 1 as indicated in the scaling bar under the Figure. For the full and clear metabolites names used in this figure, please refer to Table S5.

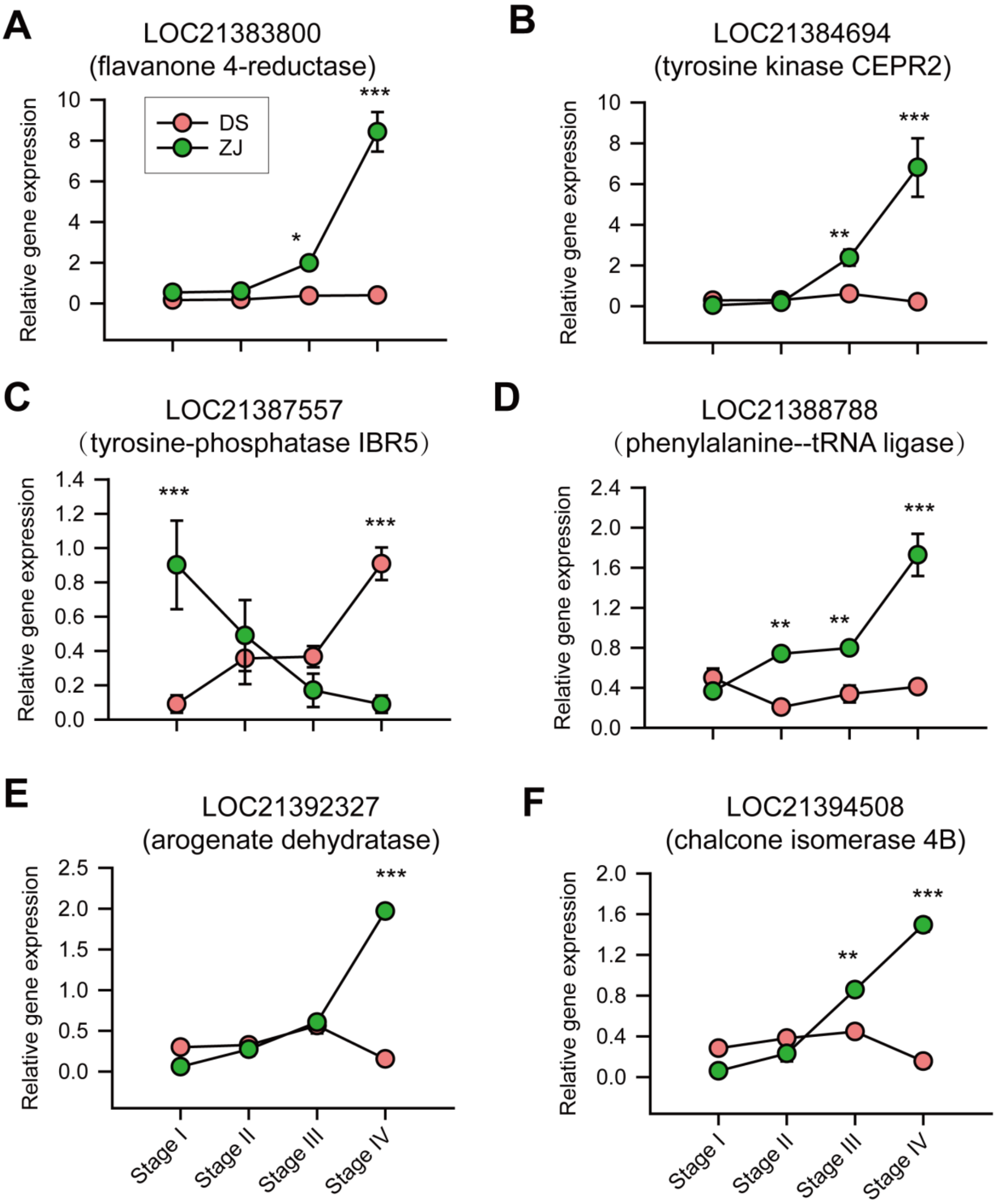

Figure S6. Differential expression analysis of the genes related to anthocyanin biosynthesis in fruits of DS and ZJ mulberry genotypes at different developmental stages. Each data point represents the mean of three different replicates (n = 3) ±SE. Symbols "*", "**", and "***" represent the significant difference level between DS and ZJ at a *P*-value < 0.05, 0.01 and 0.001, respectively, based on a Student's *t*-test. The detailed description of corresponding gene ID is referred to Table S7.

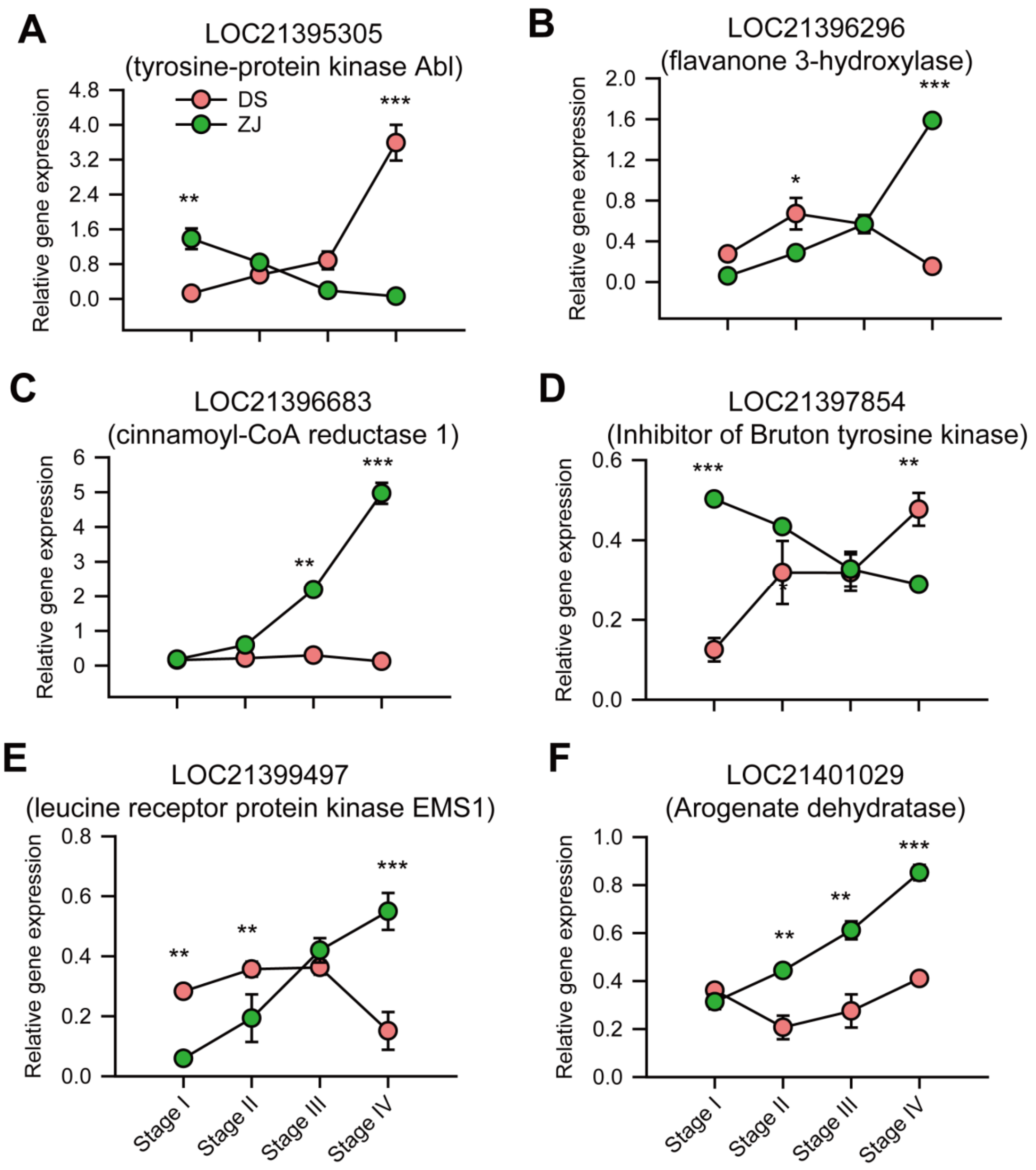

Figure S7. Differential expression analysis of the genes related to anthocyanin biosynthesis in fruits of DS and ZJ mulberry genotypes at different developmental stages. Each data point represents the mean of three different replicates (n = 3) ±SE. Symbols "*", "**", and "***" represent the significant difference level between DS and ZJ at a *P*-value < 0.05, 0.01 and 0.001, respectively, based on a Student's *t*-test. The detailed description of corresponding gene ID is referred to Table S7.

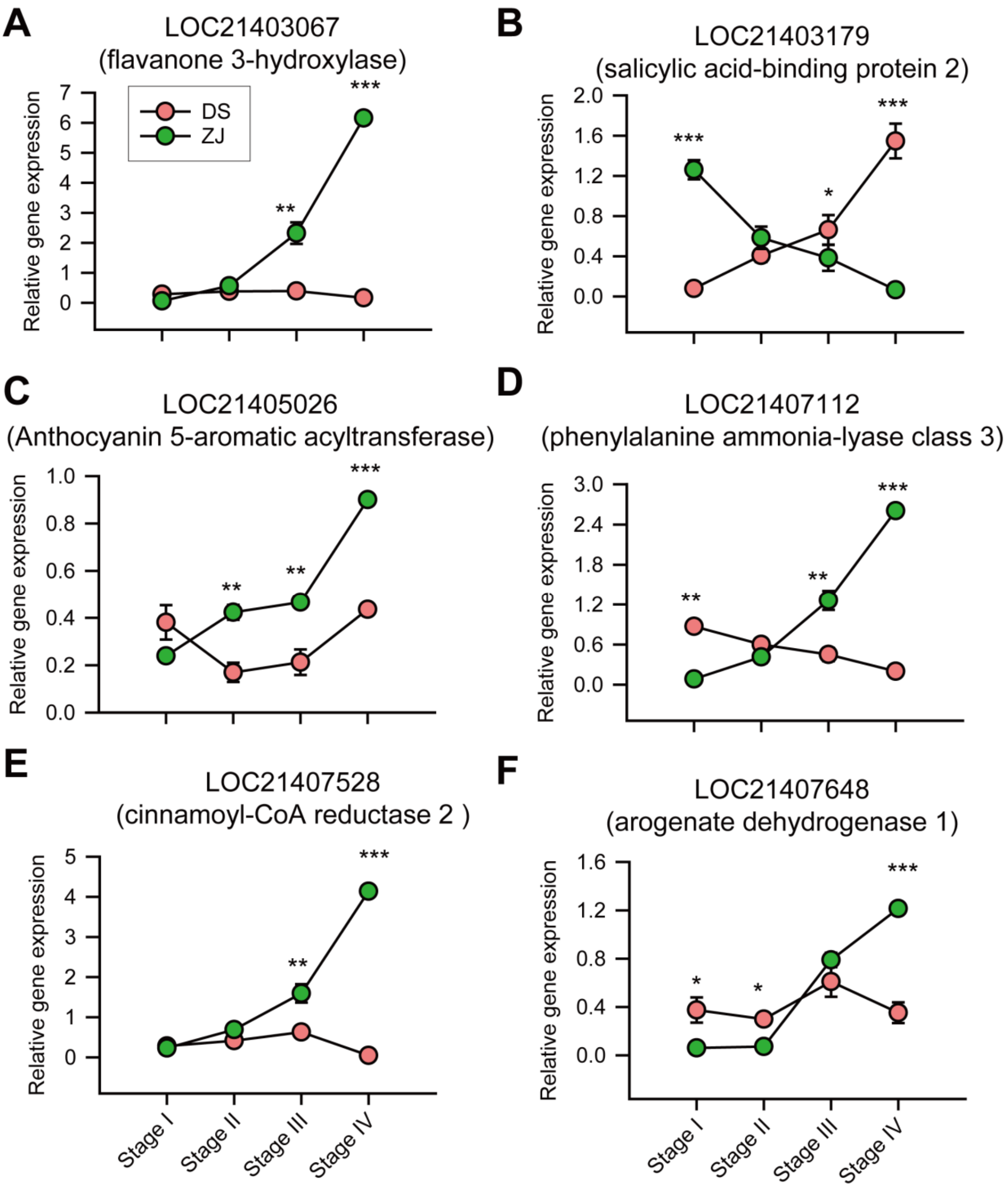

Figure S8. Differential expression analysis of the genes related to anthocyanin biosynthesis in fruits of DS and ZJ mulberry genotypes at different developmental stages. Each data point represents the mean of three different replicates (n = 3) ±SE. Symbols "*", "**", and "***" represent the significant difference level between DS and ZJ at a *P*-value < 0.05, 0.01 and 0.001, respectively, based on a Student's *t*-test. The detailed description of corresponding gene ID is referred to Table S7.

.

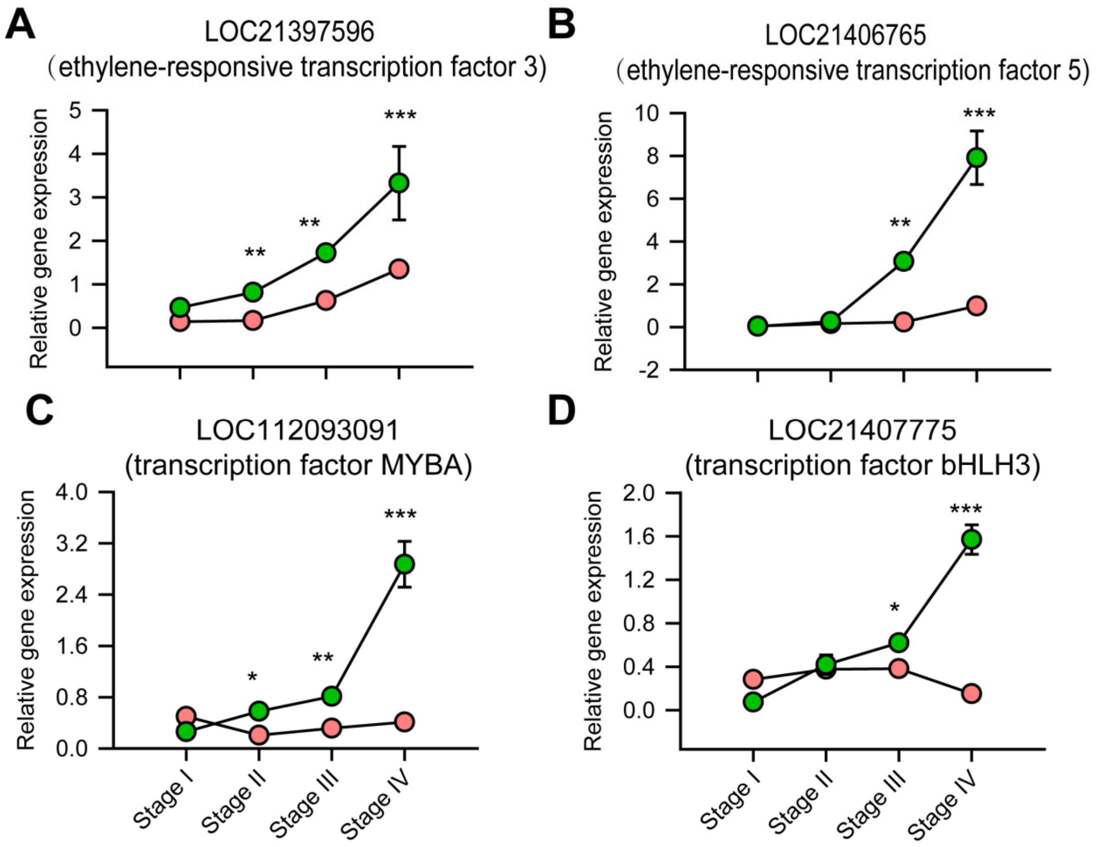

Figure S9. Differential expression analysis of the transcription factors related to anthocyanin biosynthesis in fruits of DS and ZJ mulberry genotypes at different developmental stages. Each data point represents the mean of three different replicates (n = 3) ±SE. Symbols "*", "**", and "***" represent the significant difference level between DS and ZJ at a *P*-value < 0.05, 0.01 and 0.001, respectively, based on a Student's *t*-test. The detailed description of corresponding gene ID is referred to Table S7.
