# Supplementary material for: The Ethylene Response Factor ERF5 Regulates Anthocyanin Biosynthesis in ‘Zijin’ Mulberry Fruits by Interacting with MYBA and F3H Genes
Source: Int J Mol Sci. 2022 Jul 9;23(14):7615. doi: 10.3390/ijms23147615 (PMC9318412; doi:10.3390/ijms23147615)
Supplement: Supplementary file 1 [file ijms-23-07615-s001.zip › Supp Tables.pdf]

Table S1. List of primers used in this study.

Gene ID	Primer sequence	Product size (bp)
LOC21409648-F	CACGGAGCTTAGGATTGCCA	115
LOC21409648-R	ACACCAAGAGCCAAGTGAGG	
LOC21396296-F	CATTCCCGAGATACCCACCG	131
LOC21396296-R	GTATGACCTCGGCAGGGATG	
LOC21396683-F	CGGCATCTCCCGTTACTGAT	118
LOC21396683-R	GAGGACGTGAACACCACTCG	
LOC21407528-F	ATTTTCACGGCAAACTGGGC	129
LOC21407528-R	GGCTCGTACCCGTCTTTCAA	
LOC21383800-F	AATCAGGGCGAGCTAGTGTG	110
LOC21383800-R	AGATCTTTGACGGTGGCGTT	
LOC21394508-F	AGGGCAAGACGGGAAATGAC	146
LOC21394508-R	CCTCAAAGCGCTCTCGATCT	
LOC112095175-F	AGGGCAAGACGGGAAATGAC	146
LOC112095175-R	CCTCAAAGCGCTCTCGATCT	
LOC21412324-F	TAAAGCAGCAAGCAATGGCG	117
LOC21412324-R	TGCTAGGATTGGATGTGCCA	
LOC21412880-F	GGGTCGGTCGAAAGACCATT	105
LOC21412880-R	TGTGAGTCCGCAATCACGAA	
LOC21388788-F	TGTTCGAAGCGCTAGGGAAG	140
LOC21388788-R	TTCCTCGGCTTTTTCGTCCA	
LOC21407112-F	GCCAACATTCTTGCCGTGTT	131
LOC21407112-R	GCTGCAGCCTCAATTTGACC	
LOC112094761-F	CAAGTGGCGAAGAGAGTGCT	149
LOC112094761-R	TGCATCAACGGGTAGGTAGC	
LOC21403179-F	GTCGTCGTTTTCTTGACGGC	112
LOC21403179-R	GAGTGTCCAGCCATGCATCT	
LOC21397854-F	GCAAAATCCCCAAACCACCC	117
LOC21397854-R	ACGCGTTACGCAAGAAACAC	
LOC21410011-F	AAGTTCCTGGATTGGCGGAG	122
LOC21410011-R	CACGACCTCGAAAACCGTCT	
LOC21399497-F	GCCATGTTCATGGAAGGGAGT	113
LOC21399497-R	TCTCACAGACGACAGGCAAAA	
LOC21408116-F	CAAGGGCTCCTTGCCAATCT	139
LOC21408116-R	CCAATTCCGCCATTGTTCGG	
LOC21387557-F	GGGGAGGTTTGTGGGATCTG	133
LOC21387557-R	AGCGAGAAGCGTTGTCGTAA	
LOC21384694-F	gctgggacttggggagaacg	154
LOC21384694-R	atccaacgttcccaacgcca	
LOC21395305-F	ccggcatgtcggagagatcc	126
LOC21395305-R	gcgattgtgtcgaacgccaa	
LOC21392327-F	accgctaatccagccgaagc	107
LOC21392327-R	tcgatccgatgccgtttggg	
LOC21407648-F	ccgcgcagctctacgactac	143
LOC21407648-R	ggcaaggactgtgtggcctt	
LOC21401029-F	gtctccgattgggggccaag	111

LOC21401029-R	tctctgagtctgagccgccg	
actin-F	agcacccggttctcctgact	127
actin-R	agggaaaggacggcctggat	
actin2-F	ttcagccgctcgtttgcgat	111
actin2-R	cggtgtgacggggacgacct	
LOC21406765-F	CGGAAGAGAATTGTTCGAAC	1168
LOC21406765-R	TTAATGTTACATGAAGCTGAC	
LOC21392905-F	CCAACAATTGAACTCTTTT	150
LOC21392905-R	CTCTGTTCAATCCAGCATG	
LOC21403067-F	CCCCGAGAACACCAAAGCCG[illegible]	152
LOC21403067-R	TGACGTGAAAAAATAGTCTAT	
